# Supplementary material for: Vitamin E Blocks Connexin Hemichannels and Prevents Deleterious Effects of Glucocorticoid Treatment on Skeletal Muscles
Source: Int J Mol Sci. 2020 Jun 8;21(11):4094. doi: 10.3390/ijms21114094 (PMC7312599; doi:10.3390/ijms21114094)
Supplement: Supplementary file 1 [file ijms-21-04094-s001.pdf]

Supplementary material

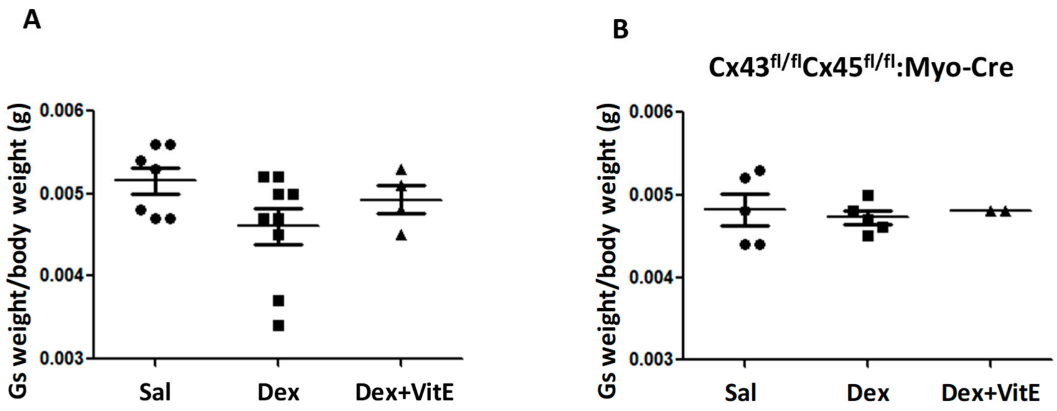

Supplementary Figure 1: Muscle weight of wild type but not Cx43/Cx45 expression deficient mice is reduce by dexamethasone. Wild type and and $Cx43^{fl/fl}Cx45^{fl/fl}$:Myo-Cre mice were treated daily during 7 days with saline (Sal) or dexamethasone (10 mg/kg Dex) or Dex 10 mg/day and fed with Vitamin E supplemented diet (Dex + VitE). At the end of the experiment mice were euthanized and body and gastrocnemius (Gs) weight was recorded. **(A)** Gs weight per body weight ratio from wild type mice. Saline = Sal; Dexamethasone = Dex; vitamin E; VitE (n sal=7; Dex=9; Dex+VitE=4). **(B)** Gs weight per body weight ratio from $Cx43^{fl/fl}Cx45^{fl/fl}$:Myo-Cre mice deficient in expression of Cx43 and Cx45 specifically in myofibers. (n sal=5; Dex=5; Dex+VitE=2).

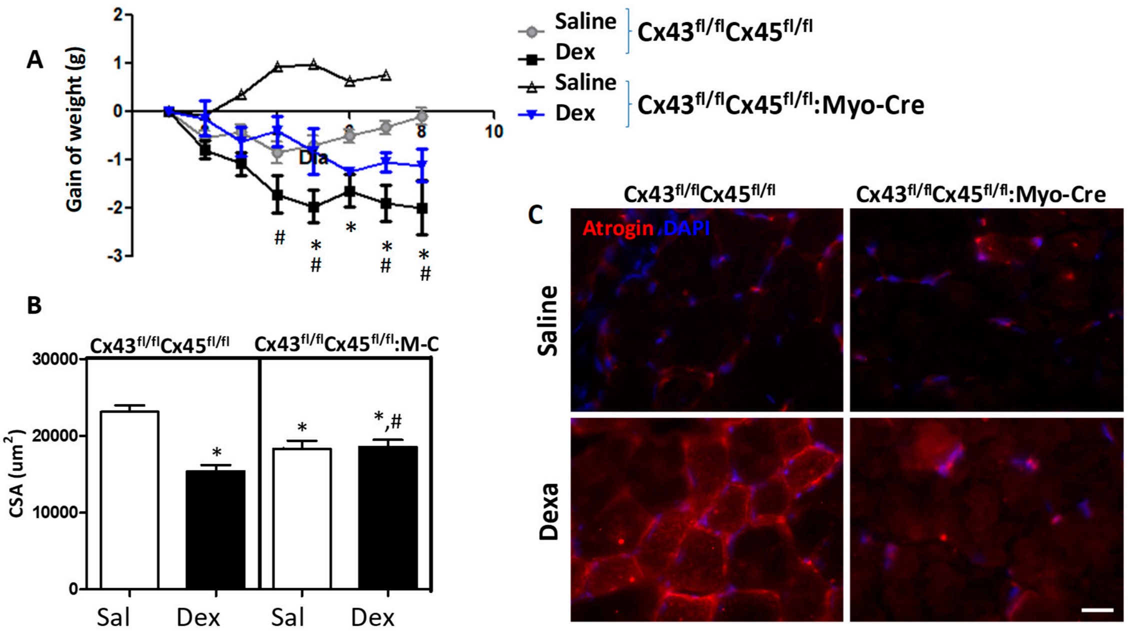

Supplementary Figure 2: **Cx43/Cx45 expression deficient mice are resistant to weight loss and muscle atrophy induced by dexamethasone.** Cx43$^{fl/fl}$Cx45$^{fl/fl}$ and Cx43$^{fl/fl}$Cx45$^{fl/fl}$:Myo-Cre mice were daily treated for 7 days with saline (Sal) or dexametahasone (Dex, 10 mg/kg Dex). **(A)** The mice weight was recorded daily. The graph shows the loss of weight relative to the start of treatment. (For Cx43$^{fl/fl}$Cx45$^{fl/fl}$ n Saline: 5; n Dex: 6 and for Cx43$^{fl/fl}$Cx45$^{fl/fl}$:Myo-Cre n Saline: 6; n Dex: 9). Significance was assessed by two-way ANOVA followed by Bonferroni post hoc test. * $p<0.05$ for Saline vs Dex Cx43$^{fl/fl}$Cx45$^{fl/fl}$ mice; # $p<0.05$ Saline Cx43$^{fl/fl}$Cx45$^{fl/fl}$ vs Dex Cx43$^{fl/fl}$Cx45$^{fl/fl}$:Myo-Cre **(B)** The cross-sectional area (CSA) of myofibers of tibialis anterior muscles was measured by off-line analysis of hematoxylin-eosin images. Five images of each muscle section were evaluated (n=3 for all groups).. The results are expressed as mean ± SEM. * $p<0.05$ different from saline Cx43$^{fl/fl}$Cx45$^{fl/fl}$ mice; # $p<0.05$ different from Dex Cx43$^{fl/fl}$Cx45$^{fl/fl}$ mice **(C)** The presence and cellular distribution of the protein-degradation marker Atrogin-1(Red) was evaluated by immunofluorescence assay in cross sections of TA muscles. (n=3 for all groups). Scale bar: 50 μm.
